# Head and Neck Metastases From Infraclavicular Primary Tumors: Analysis of 136 Cases

**DOI:** 10.1002/lary.70496

**Published:** 2026-03-15

**Authors:** Achim M. Franzen, André Buchali, Carolin Quadt, Annekatrin Coordes

**Affiliations:** ^1^ Faculty of Health Sciences Brandenburg, Joint Faculty of the University of Potsdam, Brandenburg University of Technology Cottbus‐Senftenberg and Brandenburg Medical School Potsdam Germany; ^2^ Department of Otorhinolaryngology Head and Neck Surgery, University Medical Centre Ruppin Brandenburg (Ukrb), Brandenburg Medical School Neuruppin Germany; ^3^ Department of Radiooncology University Medical Centre Ruppin Brandenburg (ukrb), Brandenburg Medical School Neuruppin Germany

**Keywords:** distant metastases, head and neck region, metastases, primary tumors

## Abstract

**Objective:**

Distant metastases in the head and neck region are rare, often posing challenges for ENT specialists and being of crucial importance in the management of infraclavicular primary tumors.

**Methods:**

The case series presented here aims to outline the relevant aetiologies and clinical presentation in the head and neck region. Aspects of diagnostics, differential diagnostics, therapy, and prognosis are discussed. A total of 136 cases (7.8%) from 1735 patients recorded in our tumor database between 2000 and 2023 were included. All cases represent patients consecutively operated on during this period.

**Results:**

The metastases of the patients (58 women, 78 men, median 64 years) were predominantly located in the cervical lymph nodes (86.8%). Organ metastases mainly affected the parotid gland (38.9%). Histologically, adenocarcinomas were most frequently diagnosed (40.4%). The primary tumors were mainly lung carcinomas (33.1%), followed by mammary and renal cell carcinomas. The clinical manifestation was indolent swelling in most cases. In 91 cases (66.9%), head and neck metastases were the first manifestation of the primary tumor. Only 7 of the cases presented with an isolated cervical metastasis.

**Conclusions:**

Localizing a metastasis in the supraclavicular neck region and/or a histological finding other than squamous cell carcinoma indicates a remote primary tumor. The considerable relevance of cervical metastases in the (initial) primary tumor diagnosis is due to their early clinical manifestation and easy biopsy accessibility. In most cases, distant cervical metastasis is an indication of advanced tumor disease.

**Level of Evidence:**

4.

## 1 Introduction

In the head and neck region, most lymph node and organ metastases originate from supraclavicular primary tumors, particularly those of the upper aerodigestive tract. Less frequently, they arise from the skin, major salivary glands, or thyroid gland. Malignant lymphomas must also be considered in the differential diagnosis of a malignant tumor.

Distant metastases from infraclavicular primary tumors are rare, with most studies reporting a frequency of approximately 1% [1]. However, the possibility of an infraclavicular primary has to be taken into consideration when malignancy is detected in a cervical lymph node or when an organ tumor in the head and neck exhibits atypical clinical or histopathological features and a local primary tumor has been ruled out. Distant metastases to the head and neck region are of crucial clinical importance in the diagnosis, therapy, and prognosis of remote primary tumors. Particularly in cases where the primary tumor is unknown, metastases in the head and neck pose a differential diagnostic challenge for ENT specialists. The clinical relevance of this topic is emphasized by the inclusion of a dedicated chapter on head and neck metastases in the fifth edition of the WHO Classification of Tumors series [2].

This case series aims to identify the most common primary tumors metastasizing to the head and neck region, to analyze their clinical presentation and disease course, and to determine diagnostic parameters relevant to differential diagnosis. Aspects relevant to therapy and prognosis are addressed to the extent that data could be generated for this purpose.

## 2 Materials and Methods

This retrospective study included 136 histologically confirmed cases (7.8%) out of 1735 patients included in the tumor database of the ENT Clinic at Ruppin‐Brandenburg University Hospital between 2000 and 2023. Excluded were metastases originating from carcinomas of the upper aerodigestive tract, skin tumors of the head and neck, salivary gland and thyroid carcinomas, malignant lymphomas, and metastases where no primary tumor could be found either in the head and neck region or infraclavicularly (CUP syndrome) after completion of the diagnostic workup, and tumors confirmed by cytology only. To specify the location of metastases in cervical lymph nodes, we distinguished between caudal/supraclavicular (levels IV and Vb), jugular (levels II and III), submandibular (level I), and nuchal lymph node stations.

Indications for biopsy included clinically suspicious cervical lymph nodes or tumors localized in the parotid or thyroid gland, skin or bone of the head and neck, and paranasal sinuses.

The procedures we performed included lymph node excisions, partial parotid resections of varying extent, hemi‐ or total thyroidectomies, endonasal sinus surgeries, excisions of skin tumors, and open bone biopsies. In all included cases, either an infraclavicular primary tumor was unknown or metastasis of an already known primary tumor had first been diagnosed by cervical biopsy. Accordingly, infraclavicular primary tumors in which cervical metastasis did not lead to the initial diagnosis of the tumor or a previously unknown metastasis were not included. Particularly, cases of disseminated, e.g., skeletal metastases were excluded.

Neck masses and parotid or thyroid tumors were typically evaluated by ultrasound, whereas preoperative computed tomography (CT) imaging was used with bone and sinunasal tumors. After receiving the histological diagnosis, further examinations were carried out to locate the primary tumor and determine the disease stage. The diagnostic measures included cross‐sectional imaging, and increasingly in recent years PET‐CT, mammography, scintigraphy and/or endoscopic examinations by other specialist disciplines (e.g., pulmonology, gastroenterology). All procedures performed in our study were diagnostic measures. Our clinic did not provide treatment for metastatic primary tumors; in particular, surgical interventions such as neck dissections were not performed.

All procedures were conducted in accordance with accepted clinical standards and the ethical principles of the Declaration of Helsinki as well as German federal law. Informed consent was obtained from all patients prior to treatment. The study was approved by the institutional review board (2024‐1‐BO). The medical records of all HNCUP patients were reviewed, including clinical history, imaging studies, operative reports, and histopathological findings.

## 3 Results

This study included 58 (43%) female and 78 (57%) male patients with a mean age of 60.1 years (median age 64 years; range 30–83 years). The metastatic tumors were predominantly (86.8%) located in the cervical lymph nodes, particularly in the caudal (supraclavicular) group. In addition, 18 organ metastases primarily affected the parotid gland and the mucosa of the paranasal sinuses (Table 1).

**TABLE 1:** Localization of 136 distant metastatic tumors in the head and neck region.

Cervical lymph nodes	118 (86.8%)	%
Supraclavicular (Level IV, Vb)	109	92.4
Jugular (Level II/III)	6	5.1
Submandibular (Level I)	1	0.8
Nuchal	2	1.7

Organs	18 (13.2%)	%
Parotid gland	7	38.9
Thyroid	3	16.7
Paranasal sinuses	4	22.2
Skull base	2	11.1
Forehead skin	1	5.6
Tongue	1	5.6

The primary tumors were mainly lung carcinomas (33.1%), breast carcinomas (23.5%), and renal cell carcinomas (20.6%) (Table 2). Histologically, adenocarcinomas were diagnosed most frequently (55 cases, 40.4%), followed by renal cell (28 cases, 20.6%), squamous cell (25 cases, 18.4%), and small cell carcinomas (19 cases, 14%). Among the lung carcinomas (45 cases, 33.1%), small cell carcinomas (19 cases, 42.2%) and squamous cell carcinomas (15 cases, 33.3%) were diagnosed most often, while adenocarcinomas and undifferentiated carcinomas were less common, with 6 and 5 cases respectively.

**TABLE 2:** Primary tumors of 136 patients with distant metastases in the head and neck region.

Primary tumors	Number	%
Bronchial tract	45	33.1
Breast	32	23.5
Gastrointestinal tract	18	13.2
Distal esophagus	5	
Stomach	4	
Colorectal	6	
Pancreas	3	
Urogenital tract	40	29.4
Kidney	28	
Bladder	5	
Testis	2	
Prostate	2	
Ovary	3	
Chest wall	1	0.7

The frequency of primary tumors that metastasized to the cervical lymph nodes corresponded to the relevance of the primary tumors (lung carcinomas 39, breast carcinomas 31, renal cell carcinomas 19). Organ metastases (18) were most frequently renal cell carcinomas (9), followed by lung carcinomas (6).

The clinical manifestation occurred according to the location of the metastases and in most cases as an indolent swelling of the affected cervical lymph nodes (105 cases), the parotid gland (6 cases), the thyroid gland (nodular goiter, 3 cases), and the forehead region (2 cases). The affected patients reported pain with enlarged cervical lymph nodes in 13 cases. Seven of those had fast‐growing metastases from small cell carcinomas in different locations. Moreover, patients reported pain in 2 out of 2 metastases located in the nuchal space. Further cases with pain in anamneses were a large parotid tumor, involvement of the paranasal sinuses (3 cases), and a submucosal metastatic tongue tumor. In addition, we observed a metastatic skin tumor of the forehead and identified two cases (mandible and sphenoid sinus) based on incidental CT findings.

When interpreting our results, it is important to distinguish between cases in which cervical biopsy was the first clinical manifestation of the tumor disease and those in which we diagnosed the recurrence of an already known tumor.

In 91 cases (66.9%), the head and neck metastasis was the first manifestation of the remote primary tumor. This applied to 36/45 lung carcinomas, 16/32 breast carcinomas, and 15/28 renal cell carcinomas.

In 35 cases, distant metastases were detected between 2 and 228 months after diagnosis of the primary tumor, with an average of 49 months. In 10 cases, the exact time interval between the primary tumor diagnosis and cervical metastasis could not be determined. Of 27 cases in which recurrences occurred at least 24 months after the primary tumor diagnosis, 12 were renal cell carcinomas, 9 were breast carcinomas, and only 1 was a lung carcinoma. One case of renal cell carcinoma received parotidectomy twice: 72 months after primary cancer we found metastatic disease in the left gland and 7 years later in the contralateral gland.

In all 91 patients in whom we diagnosed the primary tumor disease from the cervical biopsy, additional metastases were detected. Among patients with a cervical recurrence (45 cases), further metastases were detected in 27 cases, in 7 cases the cervical metastasis was the only tumor manifestation, and in 9 cases the data were incomplete. Of the cases with an isolated cervical metastasis, 6 were breast carcinomas and one was a renal cell carcinoma. The time interval between the primary tumor diagnosis and the diagnosis of the isolated metastasis was, on average, 69 months, and at least 24 months.

## 4 Discussion

This study of 136 patients is among the larger series systematically analyzing infraclavicular primaries with distant metastases to the head and neck. In addition to numerous case reports [3, 4, 5, 6] and smaller series [7], the literature includes larger series on metastases to the skull base [8, 9] and the oral cavity [10], as well as a review on cervical distant metastases [11]. Studies comparable to ours include 91 and 73 retrospective cases presented by Baum [12] and Sagheb [13].

While the frequency of distant head and neck metastases is reported as approximately 1% [1, 13], our cohort showed approximately 8%. This may reflect the extended observation period (2000–2023), a supraregional catchment of multiple organ oncology centers, and inclusion limited to histologically confirmed cases. In addition, an interdisciplinary board often recommended a biopsy of readily accessible head and neck lesions when multiple tumor‐suspect sites were present.

The age and sex distribution corresponds to comparable series [12], with expected sex differences due to the high proportion of breast cancer relative to primary head and neck cancers.

### 4.1 Target Organs and Pathways of Metastasis

Distant metastasis to the head and neck is mediated by complex, often case‐specific mechanisms. This is particularly the case in hematogenous spread to regional organs. Lymphatic drainage via the cisterna chyli and thoracic duct to the left supraclavicular fossa (Virchow's pathway) [14, 15] and vertebral plexus (Batson's pathway) [16] is well recognized. Furthermore, a direct connection from axillary nodes via the subclavian vein and internal jugular vein to cervical nodes has been described [17, 18].

Our results confirm that infraclavicular primaries particularly metastasize to caudal/supraclavicular cervical nodes, though all cervical stations may be involved [1, 11, 19, 20]. Organ metastases, in more than 83% of renal cell and lung carcinomas, were of minor significance. The parotid gland was most commonly involved in our series; the parotid gland differs significantly from other organs due to its intraglandular lymphatic structures/lymph nodes [21].

The literature indicates that skull bone metastases are most frequently observed in advanced breast, prostate, and lung cancers, with bone involvement occurring in up to 70% [9, 13, 20]. Cases of skull bone metastases as part of disseminated skeletal disease are not included. This study includes two cases with isolated bone metastases, both originating from renal cell carcinomas. Soft tissue metastases in the oral cavity are less common; about one quarter arise from lung carcinomas [10]. In the cohort studied by Baum [11] (which included patients of an oral and maxillofacial surgery clinic) orbital metastases were most common. Sinonasal metastases (4 cases in our series) most commonly originate from renal cell carcinoma [22]; the same applies to thyroid metastases [23]. Case reports document metastatic deposits across nearly all head and neck organs [6, 20, 23]. Differences between series likely reflect institutional specialization and referral patterns.

### 4.2 Primary Tumors

Our finding that bronchial, breast, and renal cell carcinomas most frequently metastasize to the head and neck is consistent with reviews and case series [1, 12, 24]. Breast cancer metastasizes to the supraclavicular nodes in up to 4.3% of cases, making it the most common cause of cervical distant metastases in women. Given the direct axillary–supraclavicular connection, there is a debate as to whether these are distant or regional metastases [1, 17, 25, 26].

Despite their quantitative significance, metastases of lung carcinomas to the supraclavicular region are relatively rare. In our cohort, small cell (*n* = 19) and squamous cell (*n* = 15) subtypes predominated, differing from others who report up to one third adenocarcinoma or large cell carcinoma metastases [1, 27, 28]. The disproportionate relevance of renal cell carcinoma for head and neck metastasis is widely reported [1, 12], with lymphatic spread via the thoracic duct and hematogenous spread via Batson's plexus commonly discussed [16]. As reported by other observers, prostate carcinoma, while the most common male malignancy, shows low rates of supraclavicular metastasis in our series [5, 29]. Colorectal carcinoma, although the third most common tumor entity overall, constituted only 4.4% of our cases, in line with other reports [1, 6, 12, 13]. As our results reveal, various other urological and gynecological tumors also metastasize to the head and neck [1, 12, 17, 30]. Virchow's classic description of gastric carcinoma metastasis to the left supraclavicular node (“Virchow's node”) [14, 15] represented 3 cases in our cohort. Contemporary literature largely comprises case reports [31].

### 4.3 Histology and Differential Diagnosis

More than 90% of metastatic tumors in the head and neck region are squamous cell carcinomas mainly originating in the upper aerodigestive tract. In our cohort, the frequency is below 20%, while adenocarcinomas predominated, consistent with other series [1, 12, 19]. Thus, histology from head and neck biopsies indicates the possible primary site when this is unknown. Nevertheless, when an adenocarcinoma is detected in a cervical lymph node, a salivary gland carcinoma should always be ruled out. In the case of small cell carcinomas, consideration should be given that they can also arise in all organs of the head and neck region [1, 32].

Otherwise, lung carcinomas are an important differential diagnosis when squamous cell metastases are detected in cervical nodes and the primary is unknown. In our experience, carcinomas of the upper aerodigestive tract rarely metastasize to caudal lymph node stations, unless in recurrence [33].

### 4.4 Clinical Manifestation

Presentation in our patients was most commonly indolent and less commonly a painful swelling of cervical nodes or an organ (e.g., parotid), in line with prior reports [12, 13]. In renal cell carcinoma, pronounced intraoperative bleeding tendency was consistently noted. The final diagnosis of our cases was based on histology/IHC.

Notably, two‐thirds of cases represented the first clinical manifestation of a previously unknown or not‐yet‐confirmed primary tumor [12, 21]. Among our three major entities, this applied to 80% of lung carcinomas and ~50% of breast and renal cell carcinomas. The diagnostic significance of cervical metastases in our cohort reflects early clinical manifestation and easier biopsy, e.g., a supraclavicular node is more accessible than a peripheral lung carcinoma. Our data support the recommendations for a thorough work‐up of unclear cervical tumors, including an appropriate IHC panel and imaging (CT/PET‐CT) within a multidisciplinary board [11, 34].

Late recurrences in our series were predominantly renal cell and breast carcinomas, highlighting their sometimes atypical course, often independent of local recurrence, second primaries, or other metastases. In contrast, lung carcinomas rarely recurred late; only one case metastasized ≥ 36 months after the primary [10, 12, 33, 35].

### 4.5 Implications for Diagnosis, Therapy Planning, and Prognosis

Comprehensive staging after histological diagnosis revealed generalized tumor involvement in all 91 first‐manifestation cases. This applied to about 80% of our cases with already known primaries. Isolated recurrent cervical metastases were mainly breast carcinomas and were characterized by long intervals since primary treatment.

Therapy determination should integrate staging, clinical parameters, and prognosis via multidisciplinary tumor boards. In generalized disease, the vast majority of our cases, curative options are generally absent. For isolated distant metastases, a curative approach including surgery may improve outcomes according to the literature [1, 11, 17, 25, 26, 27, 30]. Head and neck surgery can also provide symptom control (e.g., pain, bleeding, ulceration).

Not least for these previously prognostically limited cases, modern chemotherapies, immune, hormone and molecular genetic therapies, and checkpoint inhibitors may offer promising prospects. Actually, evidence‐based recommendations for optimal management are lacking so that the level of scientific evidence and the strength of our recommendations are low [11]. However, due to the long recruitment phase of the patients included in our study, it is not possible at this point to perform a sufficient analysis of the data from this perspective.

Treatment decisions should be made through multidisciplinary tumor boards, integrating staging results, clinical parameters, and prognostic factors. In cases of generalized disease, which constituted the vast majority of our cohort, curative treatment options were generally not available [1, 11, 17, 25, 26, 27, 30]. For such cases, which were previously considered prognostically unfavorable, modern chemotherapies, immunotherapies, hormone therapies, molecularly targeted therapies, and checkpoint inhibitors may offer promising therapeutic prospects. However, evidence‐based recommendations for optimal management are currently lacking, and the level of scientific evidence supporting treatment recommendations remains low [11]. Due to the extended recruitment period of our study cohort, a comprehensive analysis of outcomes from this therapeutic perspective is not feasible at this time.

For isolated distant metastases, a curative approach including surgery may improve outcomes according to the literature; particularly in the treatment of renal cell carcinoma, metastasis surgery plays an important role regardless of further developments in systemic therapy approaches [1, 11, 17, 25, 26, 27, 30, 35].

In small series involving heterogeneous tumor entities, beyond the resection of isolated metastases presented in our study, there is ongoing discussion regarding the potential prognostic benefit of neck dissections for cervical lymph node metastases. Indications are determined by treatment options for the primary tumor and other metastatic sites, and both the appropriateness and extent of such procedures remain controversial. In particular, surgical treatment of isolated supraclavicular metastases from breast cancer appears promising, whereas the benefit in lung cancer varies depending on histological subtype. Head and neck surgery may also provide important symptom control (e.g., for pain, bleeding, or ulceration) [11, 36, 37, 38].

Our cohort includes only cases in which clinically or radiologically detectable cervical lymph nodes were completely excised (not biopsied via needle aspiration). Extended surgical procedures in the form of neck dissection following histological diagnosis were not performed, largely based on multidisciplinary tumor board decisions. The only repeated surgical intervention involved a metastasis to the contralateral parotid gland.

## 5 Limitations

Limitations include the retrospective design and potential selection bias in a tertiary center. Restricting inclusion to histologically/IHC‐confirmed cases improves diagnostic certainty but may imply bias compared to cytology‐based studies. The two‐decade inclusion period enabled a comparatively large sample size but introduced potential heterogeneity in histopathological work‐up (evolution of IHC panels) and incomplete data on therapy and clinical course.

## 6 Conclusions

- Distant metastases accounted for about 8% of cases in our tumor database, exceeding most reports. The majority were cervical lymph node metastases (87%), primarily located in the caudal/supraclavicular neck. Organ metastases (> 80% from renal cell and lung carcinomas) were less common.
- Primary tumors were mainly bronchial, breast, and renal cell carcinomas. Histologically, adenocarcinoma and renal cell carcinoma predominated; squamous cell carcinoma comprised < 20% of our cases.
- Caudal neck localization and/or non‐squamous histology may indicate a possible infraclavicular primary.
- In two‐thirds of cases, cervical metastasis leads to the initial diagnosis of the primary tumor, based on early clinical presentation and ease of biopsy. Late recurrences were particularly associated with renal cell and breast carcinomas.
- Cervical distant metastasis is typically associated with advanced disease; only seven cases (six breast carcinomas) represented isolated late recurrences amenable to potentially curative treatment.

## Funding

The authors have nothing to report.

## Conflicts of Interest

The authors declare no conflicts of interest.

## Data Availability

The data that support the findings of this study are available on request from the corresponding author. The data are not publicly available due to privacy or ethical restrictions.
